# Serum HE4 and diagnosis of ovarian cancer in postmenopausal women with adnexal masses

**DOI:** 10.1016/j.ajog.2019.07.031

**Published:** 2020-01

**Authors:** Aleksandra Gentry-Maharaj, Matthew Burnell, James Dilley, Andy Ryan, Chloe Karpinskyj, Richard Gunu, Susan Mallett, Jon Deeks, Stuart Campbell, Ian Jacobs, Sudha Sundar, Usha Menon

**Affiliations:** a Medical Research Council Clinical Trials Unit at University College London, Institute of Clinical Trials and Methodology, University College London, London; b Department of Women’s Cancer, Institute for Women’s Health, University College London, London; c Institute of Applied Health Research, University of Birmingham, Birmingham, London; d Create Fertility, London; e Pan Birmingham Gynaecological Cancer Centre, School of Cancer Sciences, City Hospital, Birmingham, United Kingdom; f University of New South Wales, Sydney, New South Wales, Australia

**Keywords:** adnexal mass, CA125, diagnosis, human epididymis 4, ovarian cancer, ovarian neoplasm, risk of malignancy, transvaginal ultrasound, ultrasound, United Kingdom Collaborative Trial of Ovarian Cancer Screening (UKCTOCS)

## Abstract

**Background:**

Transvaginal ultrasound and serum CA125 are routinely used for differential diagnosis of pelvic adnexal mass. Use of human epididymis 4 was approved in the United States in 2011. However, there is scarcity of studies evaluating the additional value of human epididymis 4.

**Objective:**

The objective of the study was to evaluate the performance characteristics of transvaginal ultrasound, CA125, and human epididymis 4 for differential diagnosis of ovarian cancer in postmenopausal women with adnexal masses.

**Study Design:**

This was a cohort study nested within the screen arms of the multicenter randomized controlled trial, United Kingdom Collaborative Trial of Ovarian Cancer Screening, based in England, Wales, and Northern Ireland. In United Kingdom Collaborative Trial of Ovarian Cancer Screening, 48,230 women randomized to transvaginal ultrasound screening and 50,078 to multimodal screening (serum CA125 interpreted by Risk of Ovarian Cancer Algorithm with second line transvaginal ultrasound) underwent the first (prevalence) screen. Women with adnexal lesions and/or persistently elevated risk were clinically assessed and underwent surgery or follow-up for a median of 10.9 years. Banked samples taken within 6 months of transvaginal ultrasound from all clinically assessed women were assayed for human epididymis 4 and CA125. Area under the curve and sensitivity for diagnosing ovarian cancer of multiple penalized logistic regression models incorporating logCA125, log human epididymis 4, age, and simple ultrasound features of the adnexal mass were compared.

**Results:**

Of 1590 (158 multimodal, 1432 ultrasound) women with adnexal masses, 78 were diagnosed with ovarian cancer (48 invasive epithelial ovarian, 14 type I, 34 type II; 24 borderline epithelial; 6 nonepithelial) within 1 year of scan. The area under the curve (0.893 vs 0.896; *P* = .453) and sensitivity (74.4% vs 75.6% ;*P* = .564) at fixed specificity of 90% of the model incorporating age, ultrasound, and CA125 were similar to that also including human epididymis 4. Both models had high sensitivity for invasive epithelial ovarian (89.6%) and type II (>91%) cancers.

**Conclusion:**

Our population cohort study suggests that human epididymis 4 adds little value to concurrent use of CA125 and transvaginal ultrasound in the differential diagnosis of adnexal masses in postmenopausal women.

Click Supplemental Materials under article title in Contents at **ajog.org**

Serum CA125 and transvaginal ultrasound (TVS) have been used in differential diagnosis of adnexal masses in postmenopausal women for the last 4 decades. These tests are the basis of guidelines in most countries for investigation of women with symptoms suspicious of ovarian cancer (OC).^1^, ^2^, ^3^

**AJOG at a Glance**

**Why was this study conducted?**

The study was conducted to assess whether inclusion of human epididymis 4 (HE4) improves the performance of serum CA125 and transvaginal ultrasound in the differential diagnosis of ovarian cancer in postmenopausal women with adnexal masses.

**Key findings**

In 1590 women who underwent clinical assessment for an adnexal mass detected on the first screen in United Kingdom Collaborative Trial of Ovarian Cancer Screening, a model incorporating age, transvaginal ultrasound, and CA125 performed similarly to one that also included HE4. Both had high sensitivity for invasive epithelial ovarian cancer.

**What does this add to what is known?**

Our population-based study suggests that HE4 adds little value to concurrent use of CA125 and transvaginal ultrasound in the diagnosis of ovarian cancer, especially invasive epithelial disease in postmenopausal women with adnexal masses.

The 2 tests are often interpreted using models, the earliest of which, the Risk of Malignancy Index (RMI),^4^ incorporates the CA125 value, menopausal status, and simple ultrasound features. Since then, there have been numerous TVS-only models (Simple rules,^5^ LR1, LR2^6^), which include further features such as septal thickness, size of solid lesions, and Doppler flow, with most recently described ADNEX model^7^ also including CA125. These models have been extensively evaluated in secondary care settings.^8^, ^9^

In 2011, based on encouraging secondary care data, human epididymis 4 (HE4) received approval from the US Food and Drug Administration for use in women presenting with an ovarian mass.

The main advantage of HE4 is that, unlike CA125, it is not elevated in endometriosis.^10^ This led to biomarker algorithms incorporating HE4 and CA125 such as the Risk of Ovarian Malignancy Algorithm (ROMA) and more recently the Copenhagen Index.^11^ However as highlighted in both recent systematic reviews,^12^, ^13^ there are currently not enough studies estimating HE4 performance in detecting early-stage tumors in the most relevant group, postmenopausal women in this clinical scenario. In addition, there is a scarcity of studies that investigate the performance of CA125, HE4, and TVS in women presenting to primary care physicians/gynaecologists.

A dualistic pathway of invasive epithelial ovarian carcinogenesis has emerged over the past decade. Type I invasive epithelial ovarian cancers, which include low-grade serous, low-grade endometrioid, clear cell, and mucinous tumours, are slow-growing, genetically stable indolent cancers, usually diagnosed in the early stage. Type II, mainly high-grade serous cancers, which are the majority of the cancers, are aggressive, are genetically unstable usually harboring p53 mutations, and account for most of the mortality.^14^ In evaluating the role of HE4, it would be important to consider the performance in the 2 groups separately.

In the screen arms of United Kingdom Collaborative Trial of Ovarian Cancer Screening (UKCTOCS), ultrasound data on adnexal masses detected during the initial screen and banked serum samples provided an opportunity to compare models incorporating CA125, HE4, and TVS features of the adnexal mass both alone and in combination in a population-based cohort of postmenopausal women.

## Materials and Methods

### Subjects

Between 2001 and 2005, 202,638 postmenopausal women from the general population in England, Wales, and Northern Ireland were randomized to multimodal screening (MMS; n = 50,640) using serum CA125 (level I) interpreted by Risk of Ovarian Cancer Algorithm and a combination of CA125 and TVS as a second-line test (level II), TVS screening (USS; n = 50,639), or no screening (n = 101,379) as described previously.^15^, ^16^

Of 101,279 women randomized to screening, 98,308 (50,078 MMS; 48,230 USS) underwent the initial annual (prevalence) screen.^17^ Women with an abnormality underwent a repeat TVS by a senior specialist in gynecological scanning (level II scan) in the USS group and a repeat CA125 and level II scan in the MMS group. Those with a persistent abnormality underwent clinical assessment with the regional center clinical team, who arranged further investigations (tumor markers, TVS, magnetic resonance imaging/computed tomography pelvis as appropriate) and were either referred for surgery or managed conservatively. All women who underwent clinical assessment and had banked serum sample within 6 months of the scan were included in this analysis.

There were some women who had OC diagnosed within 18 months of the sample who were not included in the previously mentioned analysis because they did not undergo clinical assessment (no abnormality on screening). Serum HE4 and CA125 was assayed in those for whom a sample was available.

### CA125 and HE4 assays

CA125 values were available for all women in the MMS arm because the assay was performed as part of their screening protocol, described previously.^17^ For those in the USS group, recruitment samples were assayed for CA125 using the same generation assay (Roche Diagnostics, Burgess Hill, United Kingdom) on the Roche Cobas analyzer as used in the trial.^17^, ^18^ HE4 assay (Roche Diagnostics) was run in parallel on all the samples included in the study from both groups.

### Ultrasound scan

Annual scans (level I screen) were performed by level I (certified sonographers, trained National Health Service (NHS) midwives or doctors trained in gynaecological scanning) or level II sonographers (senior sonographers, mostly at superintendent level, gynecologists or radiologists specialized in gyncological scanning), while repeat scans following the detection of an abnormality (level II screens) were performed only by the latter.

The same model of the ultrasound machine (Kretz SA2000; Kretztechnik AG, Zipf, Austria) was used at all centers. The UKCTOCS TVS closest to diagnosis or the last scan in the year 1 screening episode for women managed conservatively was included in the analysis. Scan findings recorded on the UKCTOCS ultrasound form (Appendix A) were augmented by independent review of stored static 2-dimensional images. The features captured for each adnexal or midline mass were based on simple morphological groupings (normal, normal with inclusion cyst, unilocular, unilocular solid, multilocular, multilocular solid, solid, or not visualized) based on the International Ovarian Tumor Analysis (IOTA) definitions from 2000.^19^

### Follow-up

Follow-up for cancer notification and deaths was through NHS Digital for England and Wales and Northern Ireland Cancer Registry and Business Services Organisation, Health and Social Care Northern Ireland. Women were sent 2 postal follow-up questionnaires (the first 3–5 years after randomization, the second in April 2014).^16^

Medical notes of women diagnosed with OC (as per World Health Organization 2014 classification) were reviewed by an Outcomes Review Committee who assigned the diagnosis, histological subtype, and stage, as described previously.^16^

### Statistical analysis

The primary outcome for this analysis was primary OC diagnosed within a year of the scan.

Models were constructed using TVS, CA125, and HE4. Features used were as follows: (1) age at scan (years); or (2) TVS features, which included (a) the presence of a solid component (papillations, solid areas in cystic lesions, or entirely solid lesions) grouped as not present in either ovary; present in 1 ovary (unilateral); present in both ovaries (bilateral) (Supplemental Table 1, Appendix B); this allowed for the risk associated with bilateral lesions with a solid component to be greater than that of unilateral lesions without the constraint of doubling of risk; (b) locularity defined as no locularity present in either ovary, which included both ovaries with normal morphology, normal morphology with inclusion cyst, solid or not visualized; locularity present in either or both ovaries (ie, morphology was unilocular or multilocular, irrespective of the presence of a solid component). This grouping was done as the model parameters for separate factor levels for uni/multilocular and either/both ovaries were deemed not statistically different, and some were even counterintuitively ordered (this was the only a posteriori decision made in terms of variable creation and model inclusion); (c) ascites (milliliters); or (d) dominant volume (DV). Dominant lesion was defined as the adnexal lesion associated with the highest risk of malignancy, based on findings from our previous study in which the risk of epithelial OC was highest in multilocular solid (6.6%), then solid (3.8%), and unilocular solid (2.4%) and lowest in those with persistent normal morphology (0.07%)^20^; dominant volume was defined as a log volume of the ovary or lesion deemed dominant and not necessarily the largest; or (3) biomarker values: log values of CA125 and HE4 were used as continuous rather than categories based on cutoffs.

All continuous variables were explored for whether a statistically superior transformation existed in terms of cancer prediction as well as for collinearity. All predictors collectively comprised the full model. Subset versions (ultrasound features; ultrasound plus CA125; ultrasound plus HE4; CA125 and HE4; CA125 only; and HE4 only) were created for purposes of comparison. All were adjusted for age.

Two well-known published prediction models were also included: ROMA and RMI. A modified version (RMI-mod) of the latter was used because data on intraabdominal metastasis were not available. It was not possible to assess LR1, LR2, and ADNEX models because these were described later and data on the required ultrasound features were not prospectively collected. The CA125 and HE4 only models were adjusted for age. There was no single cutoff; instead the CA125 and HE4 cutoff varied with age.

Multiple imputation was used to account for the missing values in ovary/lesion volume and morphology (details in Appendix B). In total, all 20 imputation sets created were used in producing an overall risk prediction model using Rubin’s Rules.^21^ This was true also for all the subset models that relied upon imputed data.

The risk prediction model was estimated using a penalised maximum likelihood logistic regression method as proposed by Firth^22^ (further details in Appendix B). Ten-fold cross-validation was used to explore the performance of the prediction model (and its subsets), in which the estimation for each of the 10 subgroups, and then prediction for the excluded group, was based using all 20 imputation sets.

ROMA and RMI-mod did not require cross-validation. Receiver-operating characteristis curves were used to compare the discriminative ability of the prediction methods. Formal comparison of the area under the curve (AUCs) for each model was performed using the method of DeLong et al.^23^ Sensitivity at 90% specificity (similar to most published HE4 diagnostic studies)^12^, ^24^, ^25^ was also calculated and the McNemar test for paired binary outcomes was used to compare differences in sensitivity.

Confidence intervals for the AUCs and sensitivities were derived using the bias-corrected percentiles from the bootstrap distribution (n = 5000). The Brier score (mean squared error difference) and the Hosmer-Lemeshow test with 10 groups were used to assess model fit. Positive and negative predictive values (PPVs, NPVs) and numbers needed to treat were included for each of the models.

In addition, the NPV and PPV across a range of sensitivities and specificities were calculated. Because the prevalence of OC can increase in symptomatic patients presenting to primary care and in those referred to secondary care, the PPV and NPV were also calculated at 10% and 15% prevalence.

## Results

In this study of differential diagnosis nested within the ovarian cancer screening arms of UKCTOCS, 2086 women (171 MMS, 1915 USS) of the 98,308 (50,078 MMS; 48,230 USS) who underwent the initial screen were found to have a persistent abnormality and underwent clinical assessment. A blood sample within 6 months of the scan was available in 1611 women (158 MMS, 1453 USS). Twenty-one women were excluded because they were diagnosed with OC more than a year after the last scan because the aim was to compare performance for detection of OC within a year of the test. In women who had multiple scans, the one within 6 months of the sample was chosen for this study.

The final cohort comprised 1590 women (158 MMS, 1432 USS) with adnexal masses. Median follow-up from randomization was 10.9 years. Seventy-eight (36 MMS, 42 USS) were diagnosed with an index cancer within a year of the last scan. The latter included 48 women with invasive epithelial (14 type I and 34 type II), 24 with borderline epithelial, and 6 with nonepithelial OC. The noncases were similar to all women (n = 98,308) who underwent the prevalence screen (data not shown). Cases were older at randomization and therefore at scan (median age, 64.4 vs 60.8 years), heavier, and less likely to have used an oral contraceptive pill (Table 1).

**Table 1:** Baseline characteristics of the cohort

Variables	Median (25th to 75th centiles)	
	Cases (n = 78)	Noncases (n = 1512)
Age, y, at sample taken	64.2 (57.9–68.3)^a^^,^^c^	60.4 (55.5–65.6)^c^
Years since last period at randomization	14.3 (5.0–20.0)	12.6 (5.9–19.2)
Duration of HRT use in those who were on HRT at randomization, y	9.8 (4.0–11.0)	8.2 (5.1–12.8)
Duration of OCP use, y, in those who had used it	4 (2–10)	5 (2–10)
Miscarriages (pregnancies <6 mos	0 (0 – 1)	0 (0–1)
Children (pregnancies >6 mos), n	2 (1–3)	2 (1–3)
Height, cm	162.6 (158.0–168.0)	162.6 (157.25–167.6)
Weight, kg	72.6 (64.0–82)^c^	68.0 (60.3–76.7)^c^
	n, %	
Ethnicity		
White	77 (98.7%)	1465 (96.9%)
Black	0 (0%)	20 (1.3%)
Asian	0 (0%)	12 (0.8%)
Other	1 (1.3%)	8 (0.5%)
Missing	0 (0%)	7 (0.5%)
Hysterectomy	17 (21.8%)	441 (29.2%)
Ever use of OCP	36 (46.2%)^c^	914 (60.4%)^c^
Use of HRT at recruitment	15 (19.2%)	322 (21.3%)
Personal history of cancer^b^	6 (7.7%)	86 (5.7%)
Personal history of breast cancer	5 (6.4%)	52 (3.4%)
Maternal history of ovarian cancer	2 (2.6%)	25 (1.7%)
Maternal history of breast cancer	2 (2.6%)	90 (6.0%)

*OCP*, oral contraceptive pill; *HRT*, hormone replacement therapy.

*Gentry-Maharaj et al. HE4 in diagnosis of ovarian cancer. Am J Obstet Gynecol 2020*.

a Surrogate for age at diagnosis of cancer

b Includes those with a personal history of breast cancer

c *P* < .05.

Cases had a median CA125 of 85.0 (interquartile range [IQR], 24.1, 231.6) kU/L vs 15.3 (IQR, 11.2, 22.1) kU/L in noncases. Median HE4 was also higher, 92.6 (IQR, 65.6, 215.0) pmol/L in cases vs 55.3 (IQR, 47.1, 68.9) pmol/L in noncases. Ninety-six percent of scans (1526) used in the analysis were performed by level II ultrasonographers and 4% (64) by level I.

The median volume of the dominant adnexal mass was 29.9 mL (IQR, 0.9, 40.9) in cases and 19.4 mL (IQR, 3.9, 27.5) in noncases, and the median largest diameter was 4.4 cm (IQR, 2.2, 7.4) in cases and 3.2 cm (IQR, 2.3, 4.8) in noncases. A solid component was identified in 65.4% of cases (51 of 76) vs 33.2% of noncases (502 of 1503). A total of 42.3% of cases had a multilocular solid cyst compared with 18.4% of noncases with the inverse for multilocular cyst (12.8% of cases and 40.2% of noncases) (Table 2).

**Table 2:** Basic descriptive statistics for each risk factor by ovarian cancer by ultrasound features and biomarkers and other variables

Ultrasound features	Noncases		Ovarian cancer							
			Ovarian cancer cases (all)		Borderline epithelial ovarian cancer		Primary invasive epithelial ovarian cancer/primary peritoneal cancer		Nonepithelial cancers	
	n	%	n	%	n	%	n	%	n	%
	**1512**		**78**		**24**		**48**		**6**	
Unilocular cyst	200	13.2%	7	9.0%	3	12.5%	4	8.3%	0	0.0%
Multilocular cyst	608	40.2%	10	12.8%	4	16.7%	3	6.3%	3	50.0%
Unilocular solid cyst	191	12.6%	15	19.2%	6	25.0%	7	14.6%	2	33.3%
Multilocular solid cyst	278	18.4%	33	42.3%	11	45.8%	21	43.8%	1	16.7%
Solid mass	33	2.2%	3	3.8%	0	0.0%	3	6.3%	0	0.0%
Not seen^a^	25	1.7%	1	1.3%	0	0.0%	1	2.1%	0	0.0%
Difficult to classify/missing	42	2.8%	5	6.4%	0	0.0%	5	10.4%	0	0.0%
Persistent normal ovarian morphology^b^	135	8.9%	4	5.1%	0	0.0%	4	8.3%	0	0.0%
Midline	33	2.2%	9	11.5%	2	8.3%	5	10.4%	1	16.7%
Ascites ≥10 mL	97	6.4%	12	15.4%	0	0.0%	12	25.0%	0	0.0%

Biomarkers/other variables	Median	25th	75th	Median	25th	75th	Median	25th	75th	Median	25th	75th	Median	25th	75th
		centile			centile			centile			centile			centile	
CA125, kU/L	15.3	11.2	22.1	85.0	24.1	231.6	40.5	19.8	90.5	168.2	65.4	716.6	21.2	12.5	22.9
HE4, pmol/L	55.3	47.1	68.9	92.6	65.6	215.0	71.7	56.8	88.0	138.0	86.1	613.4	61.4	54.3	68.6
Age at scan, y	60.8	55.9	66.0	64.4	58.1	68.6	62.5	58.0	70.0	64.4	58.3	68.6	65.5	60.0	68.3
Largest diameter, mm	31.6	22.8	47.5	44.0	22.0	74.0	45.9	29.8	79.2	43.0	16.0	71.0	43.5	23.0	76.7
Dominant volume	19.4	3.9	27.5	29.9	0.9	40.9	29.6	21.2	35.8	29.9	20.1	42.3	23.8	14.9	34.8

*Gentry-Maharaj et al. HE4 in diagnosis of ovarian cancer. Am J Obstet Gynecol 2020*.

a Not seen but good view of iliac vessels (16); not seen in either ovary because of ovaries being obscured (9)

b Presence of ascites (2 women, measurements, 44, 47 mm); 1 with inclusion cyst (8.4 mm); 1 with indeterminate mass adjacent to the posterior wall of the uterus (103 × 37 × 72 mm).

Individual regressions on each predictor variable, using the MI paradigm where necessary, showed highly significant associations with OC except for locularity (Supplemental Table 2). On comparing the models, the full (ultrasound, CA125 and HE4) (AUC, 0.896) and the ultrasound plus CA125 model (AUC, 0.893) had similarly high (test of difference, *P* = .453) cross-validated AUC and similar sensitivity (75.6% vs 74.4%; *P* = .564) at fixed specificity of 90% (Table 3 and Figure).

**Table 3:** Performance characteristics of a model incorporating ultrasound, CA125, and HE4 compared with subset models and ROMA and modified RMI

Model	Specificity at 90%															*P* value^a^	Brier score
	Sensitivity	L, 95% CI	U, 95% CI	AUC	L, 95% CI	U, 95% CI	PPV	L, 95% CI	U, 95% CI	NPV	L, 95% CI	U, 95% CI	NNT	L, 95% CI	U, 95% CI		
Ultrasound plus CA125 plus HE4^b^	0.756	0.654	0.843	0.896	0.847	0.935	28.1	22.1	34.7	98.6	97.9	99.2	3.74	3.05	4.85		0.028
Ultrasound plus CA125^b^	0.744	0.638	0.833	0.893	0.844	0.933	27.8	21.8	34.3	98.6	97.8	99.1	3.80	3.09	4.95	.4527	0.029
ROMA (CA125 plus HE4)	0.692	0.582	0.789	0.854	0.8	0.9	26.3	20.5	32.9	98.3	97.4	98.9	4.06	3.26	5.39	.0025	0.0554^c^
Ultrasound plus HE4^b^	0.679	0.575	0.784	0.854	0.802	0.9	26.0	20.1	32.6	98.2	97.3	98.8	4.14	3.31	5.52	.0304	0.033
CA125^b^	0.667	0.557	0.766	0.846	0.786	0.895	25.6	19.8	32.2	98.1	97.3	98.8	4.21	3.36	5.65	.0014	0.034
RMI-mod	0.641	0.529	0.738	0.859	0.801	0.906	24.9	19.1	31.4	98	97.1	98.7	4.37	3.46	5.94	.0157	NA^d^
Ultrasound^b^	0.551	0.437	0.671	0.808	0.752	0.859	22.2	16.5	28	97.5	96.5	98.2	5.09	3.91	7.27	.0005	0.041
HE4^b^	0.538	0.43	0.656	0.799	0.738	0.851	21.8	16.2	28.3	97.4	96.5	98.2	5.21	3.99	7.52	.0001	0.038

*CI*, confidence interval; *L*, lower; *NNT*, numbers needed to treat; *NPV*, negative predictive value; *PPV*, positive predictive value; *RMI*, Risk of Malignancy Index; *RMI-mod*, RMI modified; *ROMA*, Risk of Ovarian Malignancy Algorithm; *U*, upper.

*Gentry-Maharaj et al. HE4 in diagnosis of ovarian cancer. Am J Obstet Gynecol 2020*.

a Test of difference with full model

b All models incorporate age

c Not directly comparable because the predictions are based on a population with a different ovarian cancer prevalence (ie, with a different constant term)

d Not applicable because RMI does not provide predictions on the probability scale.

**Figure:**
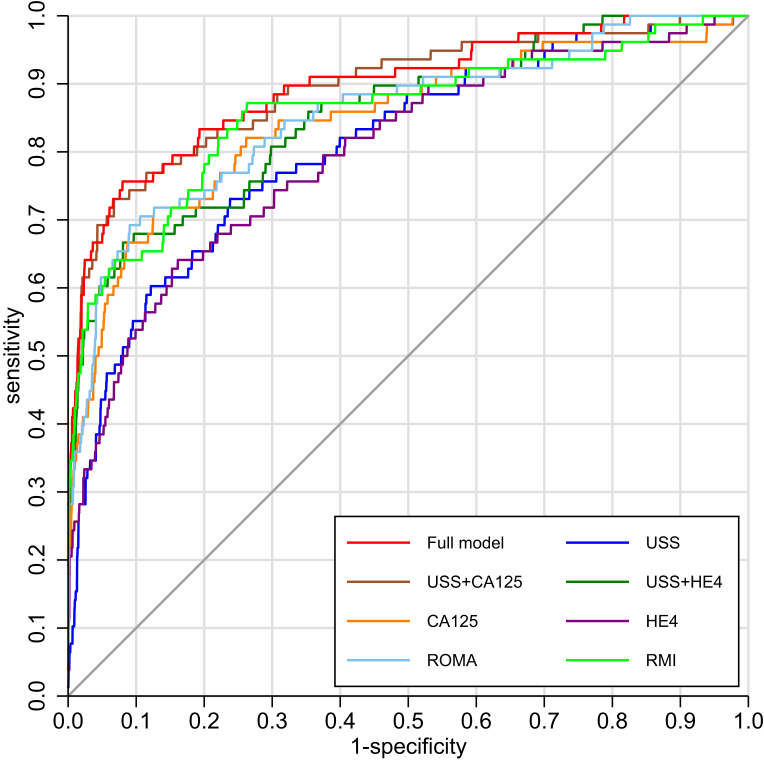
ROC curves for each risk prediction model ROC curves for the model incorporating ultrasound, age, CA125, and HE4 and subset models and ROMA and modified RMI. *HE4*, human epididymis 4; *RMI*, Risk of Malignancy Index–modified; *ROMA*, Risk of Ovarian Malignancy; *USS*, transvaginal ultrasound. *Gentry-Maharaj et al. HE4 in diagnosis of ovarian cancer. Am J Obstet Gynecol 2020*.

ROMA had an AUC of 0.854, which was statistically lower to the previously mentioned 2 models. However, its sensitivity did not differ from that of the full model (McNemar test for paired outcomes, *P* = .0956) or to the ultrasound plus CA125 model (*P* = .414). The Hosmer-Lemeshow test on the cross-validated predictions suggested the full model fit was adequate (*P* = .316), and the Supplemental Figure plots the predictions against grouped outcomes in the logit scale.

Brier scores showed that the full model had the most accurate predictions, although all scores were low. However, the Brier scores for ROMA were not directly comparable and could not be calculated for RMI-mod. The PPV of the full model and that containing ultrasound and CA125 was 28.1 and 27.8, respectively (Table 2).

For the key subgroup analysis by behavior, ROMA had similarly high sensitivity (87.5%) to the previously mentioned 2 models (89.6%, 89.6%) for invasive epithelial OC, and for type II cancers (94.1%, 94.1%, 91.2%) (Table 4). All 3 models had similar sensitivity for late-stage disease and seemed to detect more aggressive cancers. For the early stage, the ultrasound plus CA125 model had the highest sensitivity (84.2%) (Supplemental Table 3).

**Table 4:** Characteristics of the cancers detected and missed by each of the model using cutoffs derived at 90% specificity

	All ovarian cancer cases		Invasive epithelial ovarian cancer								Borderline epithelial		Nonepithelial	
			All		Type I		Type II		Number who had died 5 y after diagnosis	5 y survival rates				
Models	78		48		14		34				24		6	
Total	No	%	No	%	No	%	No	%	No	%	No	%	No	%
Detected cancers														
Ultrasound plus CA125 plus HE4^a^	59	75.6%	43	89.6%	11	78.6%	32	94.1%	18	58.1%	14	58.3%	2	33.3%
Ultrasound plus CA125^a^	58	74.4%	43	89.6%	12	85.7%	31	91.2%	18	58.1%	13	54.2%	2	33.3%
ROMA (CA125 plus HE4)	54	69.2%	42	87.5%	10	71.4%	32	94.1%	16	61.9%	11	45.8%	1	16.7%
Ultrasound plus HE4^a^	53	67.9%	37	77.1%	7	50.0%	30	88.2%	15	59.5%	14	58.3%	2	33.3%
CA125^a^	52	66.7%	39	81.3%	11	78.6%	28	82.4%	15	61.5%	12	50.0%	1	16.7%
RMI-mod	50	64.1%	39	81.3%	10	71.4%	29	85.3%	14	64.1%	10	41.7%	1	16.7%
Ultrasound^a^	43	55.1%	30	62.5%	8	57.1%	22	64.7%	12	60.0%	11	45.8%	2	33.3%
HE4^a^	42	53.8%	35	72.9%	6	42.9%	29	85.3%	14	60.0%	6	25.0%	2	33.3%
Missed cancers														
Ultrasound plus CA125 plus HE4^a^	19	24.4%	5	10.4%	3	21.4%	2	5.9%	0	100.0%	10	41.7%	4	66.7%
Ultrasound plus CA125^a^	20	25.6%	5	10.4%	2	14.3%	3	8.8%	0	100.0%	11	45.8%	4	66.7%
ROMA (CA125 plus HE4)	24	30.8%	6	12.5%	4	28.6%	2	5.9%	3	50.0%	13	54.2%	5	83.3%
Ultrasound plus HE4^a^	25	32.1%	11	22.9%	7	50.0%	4	11.8%	3	72.7%	10	41.7%	4	66.7%
CA125^a^	26	33.3%	9	18.8%	3	21.4%	6	17.6%	4	55.6%	12	50.0%	5	83.3%
RMI-mod	28	35.9%	9	18.8%	4	28.6%	5	14.7%	5	44.4%	14	58.3%	5	83.3%
Ultrasound^a^	35	44.9%	18	37.5%	6	42.9%	12	35.3%	7	61.1%	13	54.2%	4	66.7%
HE4^a^	36	46.2%	13	27.1%	8	57.1%	5	14.7%	5	61.5%	18	75.0%	5	83.3%

*HE4*, human epididymis 4; *RMI*, Risk of Malignancy Index; *RMI-mod*, RMI modified; *ROMA*, Risk of Ovarian Malignancy Algorithm*.*

*Gentry-Maharaj et al. HE4 in diagnosis of ovarian cancer. Am J Obstet Gynecol 2020*.

a All models incorporate age.

The NPV and PPV of the full model and that containing ultrasound and CA125 did not vary significantly across a range of sensitivities and specificities (Supplemental Table 5) or OC prevalence (Supplemental Table 6).

Thirteen women (12 invasive, 1 borderline epithelial) who developed ovarian cancer within 1.5 years of the sample were not included in this analysis because they were not referred for clinical assessment. Samples were available in 7 of 8 women with a normal TVS. HE4 levels were elevated (>128 pmol/L) in 1 woman and CA125 (>30 kU/L) in 2 (Supplemental Table 4). Samples were not available for the HE4 assay in the 5 women (4 invasive, 1 borderline) who had a normal CA125 (<30 kU/L) screen) and no TVS.

## Comment

### Principal findings

Despite encouraging preliminary data on HE4, our results suggest that in postmenopausal women its role in differential diagnosis of adnexal masses is limited. It adds little value to the concurrent use of serum CA125 and simple ultrasound features, either for detection of OC overall or the invasive epithelial OC subgroup.

### Results in context

Our results suggesting CA125 and ultrasound have the best performance are in keeping with the US (American College of Obstetricians and Gynecologists)^2^ and Scottish^3^ referral guidelines for women with symptoms/adnexal mass. Neither biomarkers (CA125 or HE4) alone nor TVS alone performed well, bringing into question the sequence of tests (CA125 followed by TVS) in the UK National Institute for Health and Care Excellence guidance for detection of ovarian cancer in primary care, especially in women older than 50 years. Of note, the guidance does include repeat CA125 in women with persistent symptoms.^1^

ROMA, which combines HE4 and CA125, had high sensitivity for invasive epithelial and type II OCs (mostly high-grade serous), similar to that of the ultrasound, CA125, and HE4 model, supporting its use as an alternative in settings where TVS may not be readily available. It is currently used in clinical practice in the United States and some private clinics in the United Kingdom.^26^

In our study, HE4 alone performed less well (lower sensitivity and AUC) compared with CA125 alone, although the differences were not significant. The sensitivity of HE4 alone in postmenopausal women was lower than the pooled sensitivity (77%; 95% confidence interval, 0.72–0.81) at similar specificity (91%; 95% confidence interval, 0.89–0.94) reported in the most recent systematic review.^12^ Key contributing factors were the meta-analysis using a variety of tests (enzyme-linked immunosorbent assay, chemiluminescent microparticle immuno assay), blood and serum values, varying marker thresholds in different studies, and unavailability of raw data, resulting in categorizations as in the papers. Equally important was the use of hospital cohorts with high OC prevalence (15–59%) compared with 5% in our population-based cohort.

Because our cohort includes women who might never have presented with a symptomatic adnexal mass, we calculated the PPV and NPV at higher OC prevalence of 10%, which may be closer to that in the primary care population, and 15%, similar to prevalence in secondary care referral clinics in the United Kingdom. While both increased with higher disease prevalence, there was no additive value of HE4. It would therefore be difficult to justify including HE4 in triage unless TVS was not locally available.

It is also important to note that no definitive conclusions can be drawn that the model including HE4 has lower sensitivity for differential diagnosis of early-stage ovarian cancer because the numbers involved are too small.

### Clinical and research implications

It is likely that symptom awareness campaigns will result in women presenting with masses midway between those described in our population cohort and those currently seen in secondary care and rapid access clinics.^27^ If 12–50%^28^, ^29^ of the 10 million UK women aged >55 years^30^ presented with alarm symptoms every year, this could equate to 1.2 million women requiring tests, with a significant proportion being referred to secondary care. Therefore, a simple cost-effective protocol that is easy to implement is critical, given the widespread OC symptom awareness campaigns.

Based on our findings, the additional cost and logistics of performing HE4 is not justified because the positive and negative predictive values of including HE4 with ultrasound and CA125 were similar. A prospective study to confirm these findings is needed. In the United Kingdom, one such study is the Refining Ovarian Cancer Test accuracy Scores,^27^ with data collection underway.

### Strengths and limitations

The key strength of our study is the minimization of selection bias seen in previous diagnostic studies through the use of a prospective-specimen-collection, retrospective-blinded-evaluation design.^31^ We included serum samples collected from all women with adnexal masses (population cohort) detected on the initial ovarian cancer screen of 98,308 UKCTOCS participants from the general population. Completeness of follow-up through postal questionnaires and electronic health record linkage to cancer and death registry to ascertain diagnosis of OC in these women was 98.9%. OC diagnosis was independently confirmed by an Outcomes Review Committee.^16^ While the numbers reflect the low incidence of OC, they are likely to be more representative of the proportion of women with OC who are seen in primary care as compared with secondary care case control sets. General practitioners in the United Kingdom would expect to see a woman with OC every 5 years and typically carry out 25,000 consultations per 1 case of OC.^32^

The study of 1590 women with adnexal masses (78 ovarian cancers, 1512 controls) had 90% power to detect a difference in the AUC of 10% between the full model (AUC, 0.896) and another model. The small numbers, however, precluded a split of the data into training and test set. Instead, 10-fold cross-validation of the models limited the upward bias of prediction using the same data. We used a bespoke multiple imputation model using chained equations for missing data, methodology designed to reduce the known small-sample bias of maximum likelihood, and use of age rather than menopausal status (as in ROMA, RMI) whose definition could be challenging.

There remains an element of selection bias because 12 women with invasive epithelial OC (4 MMS, 8 USS) were not detected on the initial screen and therefore did not undergo clinical assessment. However, we were able to assay the samples in 7 of these cases. Additionally, self-selection resulted in UKCTOCS participants being healthier and less deprived than the general population.^33^

Of note, the cohort includes women with adnexal masses that might never become symptomatic. TVS alone in our cohort did not perform as well as in the IOTA group studies.^8^, ^9^ A number of factors are likely to contribute to this difference: the latter conducted in secondary care symptomatic patients with larger adnexal masses (median diameter of 10.6 cm in stage I and 8.5 cm in stage II–IV^7^ vs 4.4 cm in our study), more advanced disease,^7^ additional features including Doppler, and TVS performed by IOTA-trained sonographers.^34^

Of note, the majority of our scans (96%) were performed by senior NHS ultrasonographers. Similar variables were captured as those in the NHS, making our findings generalizable to this setting. Capture of data using earlier IOTA definitions^19^ prevented evaluation of more recent IOTA models (which include size of the solid component, number of papillations, etc).

Finally, it was not possible using this study design to evaluate the performance of HE4 as a first-line test because all the women were triaged to clinical assessment based on CA125 or TVS findings. Separate analysis of performance of HE4 as a first-line screening test in UKCTOCS is underway using a different sample set.

Our population-based study suggests that in differential diagnosis of ovarian cancer, especially invasive epithelial disease in postmenopausal women with adnexal masses, HE4 adds little value to the concurrent use of CA125 and transvaginal ultrasound.

## Figures and Tables

**Supplemental Figure:**
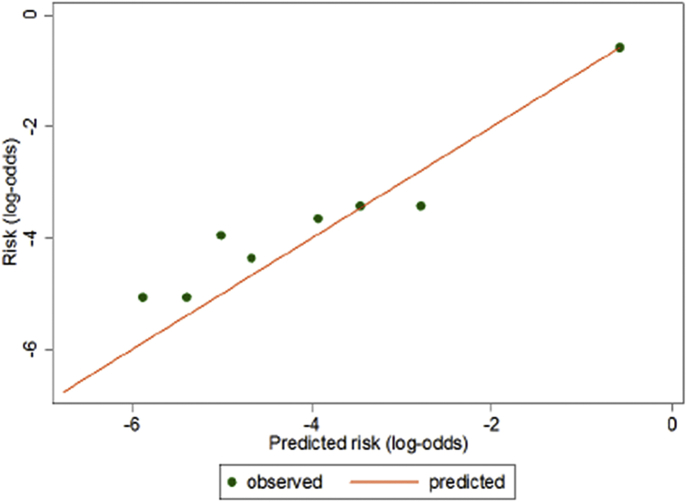
Predicted risk vs risk in logit scale: assessment of model fit (online only) *Gentry-Maharaj et al. HE4 in diagnosis of ovarian cancer. Am J Obstet Gynecol 2020*.

**Supplemental Table 1:** Basic descriptive statistics for each risk factor by ovarian cancer ultrasound features

Morphology	Noncases		Ovarian cancer	
	n	%	n	%
Unilateral solid	320	21.3%	35	46.1%
Bilateral solid	21	1.4%	11	14.5%
Unilateral unilocular	40	2.7%	3	4.0%
Bilateral unilocular	651	43.3%	30	39.5%
Unilocular and multilocular	109	7.3%	4	5.3%
Unilateral multilocular	136	9.1%	12	15.8%
Bilateral multilocular	21	1.4%	11	14.5%
Any locularity	1281	85.2%	70	92.1%

*Gentry-Maharaj et al. HE4 in diagnosis of ovarian cancer. Am J Obstet Gynecol 2020*.

**Supplementary Table 2:** Univariable regression results for all risk factors and results of the model incorporating ultrasound, age, CA125, and HE4

Univariable regression results for all risk factors	β	SE	Odds ratio^a^	Lower 95% CI	Upper 95% CI	*P* value
Risk factor						
Unilateral solid	1.448	0.2545	4.256	2.585	7.009	< .0001
Bilateral solid	3.069	0.4076	21.531	9.685	47.863	
Locularity	0.404	0.3700	1.498	0.725	3.094	.2750
Log CA125	1.463	0.1250	4.318	3.379	5.516	< .0001
Log HE4	1.990	0.2116	7.315	4.832	11.075	< .0001
Ascites, mL	0.040	0.0099	1.041	1.021	1.061	< .0001
Age at scan, y	0.060	0.0182	1.062	1.025	1.100	.0010
Midline	1.795	0.3875	6.019	2.816	12.864	< .0001
Log dominant volume	0.524	0.0764	1.688	1.453	1.961	< .0001
Results of an age-adjusted model incorporating ultrasound, CA125, and HE4						
Presence of a solid component (unilateral)	1.294	0.3254	3.649	1.928	6.906	.0001
Presence of a solid component (bilateral)	1.601	0.6358	4.959	1.426	17.248	
Locularity	1.419	0.5962	4.135	1.285	13.303	.0170
Log CA125	1.346	0.1805	3.841	2.696	5.470	< .0001
Log HE4	0.976	0.3323	2.654	1.383	5.090	.0030
Ascites, mL	0.029	0.0176	1.030	0.995	1.066	.0980
Age at scan, y	0.041	0.0237	1.042	0.994	1.091	.0850
Midline	1.292	0.6264	3.640	1.066	12.426	.0390
Log dominant lesion volume	0.280	0.0972	1.324	1.094	1.601	.0040
Constant	–19.040	2.3134				

*CI*, confidence interval; *HE4*, human epididymis 4.

*Gentry-Maharaj et al. HE4 in diagnosis of ovarian cancer. Am J Obstet Gynecol 2020*.

a equals exp(β).

**Supplementary Table 3:** Stage distribution of the cancers detected and missed by each of the models

	Primary invasive epithelial ovarian cancer					Borderline epithelial cancers					
Models	Overall, n	Early stage		Late stage		Overall, n	Early stage		Late stage		Nonepithelial
Total	48	19		29		24	21		3		6
Detected cancers	No	No	%	No	%	No	No	%	No	%	No
Ultrasound plus CA125 plus HE4^a^	43	15	78.9	28	96.6	14	11	52.4	3	100.0	2
Ultrasound plus CA125^a^	43	16	84.2	27	93.1	13	10	47.6	3	100.0	2
ROMA (CA125 plus HE4)	42	14	73.7	28	96.6	11	8	38.1	3	100.0	1
Ultrasound plus HE4^a^	37	14	73.7	23	79.3	14	11	52.4	3	100.0	2
CA125^a^	39	14	73.7	25	86.2	12	9	42.9	3	100.0	1
RMI-mod	39	14	73.7	25	86.2	10	7	33.3	3	100.0	1
Ultrasound^a^	30	14	73.7	16	55.2	11	8	38.1	3	100.0	2
HE4^a^	35	10	52.6	25	86.2	6	5	23.8	1	33.3	2
Missed cancers											
Ultrasound plus CA125 plus HE4^a^	5	4	21.1	1	3.4	10	10	47.6	0	0.0	4
Ultrasound plus CA125^a^	5	3	15.8	2	6.9	11	11	52.4	0	0.0	4
ROMA (CA125 plus HE4)	6	5	26.3	1	3.4	13	13	61.9	0	0.0	5
Ultrasound plus HE4^a^	11	5	26.3	6	20.7	10	10	47.6	0	0.0	4
CA125^a^	9	5	26.3	4	13.8	12	12	57.1	0	0.0	5
RMI-mod	9	5	26.3	4	13.8	14	14	66.7	0	0.0	5
Ultrasound^a^	18	5	26.3	13	44.8	13	13	61.9	0	0.0	4
HE4^a^	13	9	47.4	4	13.8	18	16	76.2	2	66.7	5

Early stage includes stages I and II. *HE4*, human epididymis 4; *RMI*, Risk of Malignancy Index; *ROMA*, Risk of Ovarian Malignancy Algorithm*.*

*Gentry-Maharaj et al. HE4 in diagnosis of ovarian cancer. Am J Obstet Gynecol 2020*.

a All models incorporate age.

**Supplemental Table 4:** HE4, CA125, and clinical characteristics of the 7 women from the USS group not detected through screening

Patient	HE4, pmol/L	CA125, kU/L	Time from sample to diagnosis, y	Primary invasive ovarian cancer	
				Histological subtype	Stage
1	46.91	15.57	1.4	High-grade serous	IIIc
2	59.63	8.94	1.2	High-grade serous	IIIc
3	63.06	17.92	0.9	High-grade serous	IV
4	100.0	64.89	1.1	High-grade serous	IIIc
5	51.98	30.87	1.1	Carcinosarcoma	IIIc
6	247.5	19.98	1.2	Carcinoma, NOS	IV
7	126.1	25.44	0.5	Carcinoma, NOS	IV

*HE4*, human epididymis 4; *USS*, transvaginal ultrasound.

*Gentry-Maharaj et al. HE4 in diagnosis of ovarian cancer. Am J Obstet Gynecol 2020*.

**Supplemental Table 5:** Performance of the models by varying sensitivity and specificity

Model	Specificity	Sensitivity	L, 95% CI	U, 95% CI	PPV	L, 95% CI	U, 95% CI	NPV	L, 95% CI	U, 95% CI
Ultrasound plus CA125 plus HE4^a^	80%	83.3	75.0	91.7	17.7	13.9	22.0	98.9	98.2	99.4
Ultrasound plus CA125^a^		80.8	72.2	89.3	17.3	13.5	21.5	98.8	98.0	99.3
ROMA (CA125 plus HE4)		73.1	63.1	83.1	15.9	12.3	20.1	98.3	97.4	98.9
Ultrasound plus HE4^a^		71.8	61.6	82.0	15.6	12.0	19.8	98.2	97.3	98.9
CA125^a^		73.1	62.8	83.3	15.9	12.3	20.1	98.3	97.4	98.9
RMI-mod		76.9	66.8	87.1	16.5	12.9	20.8	98.5	97.7	99.1
Ultrasound^a^		65.4	54.7	76.1	14.4	11.0	18.6	97.8	96.8	98.6
HE4^a^		65.4	54.4	76.4	14.4	11.0	18.6	97.8	96.8	98.6
Ultrasound plus CA125 plus HE4^a^	85%	78.2	69.3	87.2	21.2	16.6	26.4	98.7	97.9	99.2
Ultrasound plus CA125^a^		78.2	69.2	87.2	21.2	16.6	26.4	98.7	97.9	99.2
ROMA (CA125 plus HE4)		71.8	61.6	82.0	19.8	15.3	24.9	98.3	97.5	98.9
Ultrasound plus HE4^a^		67.9	57.4	78.5	18.9	14.5	24.0	98.1	97.2	98.8
CA125^a^		71.8	61.6	82.0	19.8	15.3	24.9	98.3	97.5	98.9
RMI-mod		70.5	59.7	81.3	19.6	15.1	24.7	98.2	97.4	98.9
Ultrasound^a^		61.5	50.3	72.7	17.5	13.2	22.5	97.7	96.8	98.5
HE4^a^		60.3	48.6	71.9	17.2	12.9	22.1	97.6	96.7	98.4
Ultrasound plus CA125 plus HE4^a^	90%	75.6	66.2	85.1	28.1	22.1	34.7	98.6	97.9	99.2
Ultrasound plus CA125^a^		74.4	64.8	83.9	27.8	21.8	34.3	98.6	97.8	99.1
ROMA (CA125 plus HE4)		69.2	58.6	79.9	26.3	20.5	32.9	98.3	97.4	98.9
Ultrasound plus HE4^a^		67.9	57.3	78.6	26.0	20.1	32.6	98.2	97.3	98.8
CA125^a^		66.7	55.9	77.4	25.6	19.8	32.2	98.1	97.3	98.8
RMI-mod		64.1	53.2	75.0	24.9	19.1	31.4	98.0	97.1	98.7
Ultrasound^a^		55.1	43.3	67.0	22.2	16.5	28.7	97.5	96.5	98.2
HE4^a^		53.8	42.3	65.3	21.8	16.2	28.3	97.4	96.5	98.2
Ultrasound plus CA125 plus HE4^a^	95%	66.7	55.7	77.6	40.6	32.0	49.7	98.2	97.4	98.8
Ultrasound plus CA125^a^		69.2	58.4	80.1	41.5	33.0	50.5	98.4	97.6	98.9
ROMA (CA125 plus HE4)		61.5	49.6	73.5	38.7	30.1	47.9	98.0	97.1	98.6
Ultrasound plus HE4^a^		60.3	48.7	71.8	38.2	29.6	47.4	97.9	97.0	98.6
CA125^a^		53.8	41.0	66.7	35.6	27.0	44.9	97.6	96.6	98.3
RMI-mod		59.0	47.7	70.3	37.7	29.1	46.9	97.8	96.9	98.5
Ultrasound^a^		43.6	30.3	56.9	30.9	22.4	40.4	97.0	96.0	97.8
HE4^a^		38.5	26.8	50.1	28.3	20.0	37.9	96.8	95.7	97.6
Ultrasound plus CA125 plus HE4^a^	80%	81.5	66.0	93.1	18.1	14.2	22.6	98.7	97.9	99.3
Ultrasound plus CA125^a^		81.0	67.6	93.3	17.8	13.9	22.2	98.7	97.9	99.3
ROMA (CA125 plus HE4)		68.2	60.3	90.2	13.1	10.2	16.4	98.6	97.7	99.2
Ultrasound plus HE4^a^		70.2	57.7	84.7	12.1	9.4	15.2	98.5	97.6	99.1
CA125^a^		74.6	52.6	87.5	13.9	10.8	17.5	98.6	97.7	99.2
RMI-mod		78.0	48.7	84.1	15.7	12.3	19.7	98.7	97.8	99.2
Ultrasound^a^		60.5	43.3	76.8	9.4	7.3	11.9	98.3	97.2	99.0
HE4^a^		59.4	47.0	71.2	9.2	7.1	11.6	98.2	97.2	99.0
Ultrasound plus CA125 plus HE4^a^	85%	74.2	49.8	86.8	14.5	11.4	18.0	98.9	98.2	99.5
Ultrasound plus CA125^a^		70.8	57.7	87.8	13.0	10.2	16.2	98.9	98.1	99.4
ROMA (CA125 plus HE4)		65.4	37.3	79.1	10.8	8.5	13.5	98.8	97.9	99.4
Ultrasound plus HE4^a^		64.7	36.2	73.2	11.0	8.6	13.8	98.8	97.9	99.4
CA125^a^		61.4	41.0	77.0	10.2	7.9	12.7	98.7	97.8	99.3
RMI-mod		74.4	39.6	80.6	14.6	11.5	18.2	98.9	98.2	99.5
Ultrasound^a^		53.4	40.0	69.9	8.6	6.7	10.8	98.5	97.5	99.2
HE4^a^		51.8	35.3	64.3	8.3	6.5	10.4	98.5	97.4	99.2
Ultrasound plus CA125 plus HE4^a^	90%	64.5	38.6	80.4	11.5	9.1	14.3	99.2	98.4	99.6
Ultrasound plus CA125^a^		60.3	43.2	79.3	10.4	8.2	13.0	99.1	98.3	99.6
ROMA (CA125 plus HE4)		41.7	19.5	68.6	8.1	6.4	10.2	98.9	97.9	99.5
Ultrasound plus HE4^a^		48.5	30.9	67.6	8.2	6.5	10.3	98.9	97.9	99.5
CA125^a^		45.9	28.9	69.3	7.9	6.2	9.9	98.9	97.8	99.5
RMI-mod		43.0	19.1	77.7	7.5	5.9	9.4	98.8	97.6	99.5
Ultrasound^a^		41.7	25.3	54.8	7.4	5.8	9.2	98.7	97.5	99.5
HE4^a^		39.0	21.5	57.5	7.0	5.5	8.8	98.7	97.4	99.4
Ultrasound plus CA125 plus HE4^a^	95%	40.6	19.5	68.7	7.6	6.0	9.5	99.4	98.4	99.8
Ultrasound plus CA125^a^		42.1	10.0	59.5	7.8	6.2	9.7	99.4	98.4	99.8
ROMA (CA125 plus HE4)		18.7	5.1	40.9	6.0	4.7	7.4	98.9	97.1	99.7
Ultrasound plus HE4^a^		31.5	21.6	36.0	6.7	5.3	8.3	99.2	97.9	99.8
CA125^a^		30.2	2.0	45.1	6.6	5.2	8.2	99.1	97.8	99.8
RMI		18.6	6.9	43.7	5.7	4.5	7.1	98.6	96.4	99.6
Ultrasound^a^		28.8	2.2	42.5	6.4	5.1	8.0	99.1	97.7	99.8
HE4^a^		21.4	4.7	41.1	5.9	4.6	7.3	98.8	96.9	99.7

*CI*, confidence interval; *L*, lower; *NPV*, negative predictive value; *PPV*, positive predictive value; *RMI*, Risk of Malignancy Index; *ROMA*, Risk of Ovarian Malignancy Algorithm; *U*, upper.

*Gentry-Maharaj et al. HE4 in diagnosis of ovarian cancer. Am J Obstet Gynecol 2020*.

a All models incorporate age.

**Supplemental Table 6:** Performance of the models (at fixed specificity of 90%) by different prevalence of ovarian cancer

Model	Prevalence of ovarian cancer	Sensitivity	L, 95% CI	U, 95% CI	PPV	L, 95% CI	U, 95% CI	NPV	L, 95% CI	U, 95% CI
Ultrasound plus CA125 plus HE4^a^	10%	75.6	65.4	84.3	45.7	40.9	50.6	97.1	95.7	98.0
Ultrasound plus CA125^a^		74.4	63.8	83.3	45.3	40.4	50.3	96.9	95.6	97.9
ROMA (CA125 plus HE4)		69.2	58.2	78.9	43.5	38.4	48.8	96.3	95	97.4
Ultrasound plus HE4^a^		67.9	57.5	78.4	43.1	37.9	48.4	96.2	94.8	97.2
CA125^a^		66.7	55.7	76.6	42.6	37.4	48	96	94.7	97.1
RMI-mod		64.1	52.9	73.8	41.6	36.3	47.2	95.8	94.4	96.8
Ultrasound^a^		55.1	43.7	67.1	38	32.3	44.1	94.8	93.4	95.9
HE4^a^		53.8	43.0	65.6	37.5	31.7	43.6	94.6	93.2	95.7
Ultrasound plus CA125 plus HE4^a^	15%	75.6	65.4	84.3	57.2	52.3	61.9	95.4	93.4	96.9
Ultrasound plus CA125^a^		74.4	63.8	83.3	56.8	51.8	61.6	95.2	93.2	96.7
ROMA (CA125 plus HE4)		69.2	58.2	78.9	55.0	49.7	60.2	94.3	92.2	95.9
Ultrasound plus HE4^a^		67.9	57.5	78.4	54.6	49.2	59.8	94.1	92	95.7
CA125^a^		66.7	55.7	76.6	54.1	48.6	59.4	93.9	91.8	95.4
RMI-mod		64.1	52.9	73.8	53.1	47.5	58.6	93.4	91.3	95
Ultrasound^a^		55.1	43.7	67.1	49.3	43.1	55.6	91.9	89.9	93.6
HE4^a^		53.8	43.0	65.6	48.8	42.4	55.1	91.7	89.7	93.4

*CI*, confidence interval; *HE4*, human epididymis 4; *L*, lower; *RMI*, Risk of Malignancy Index; *RMI-mod*, RMI modified; *ROMA*, Risk of Ovarian Malignancy Algorithm; *U*, upper.

*Gentry-Maharaj et al. HE4 in diagnosis of ovarian cancer. Am J Obstet Gynecol 2020*.

a All models incorporate age.
