# Supplementary material for: Serum HE4 and diagnosis of ovarian cancer in postmenopausal women with adnexal masses
Source: Am J Obstet Gynecol. 2020 Jan;222(1):56.e1–56.e17. doi: 10.1016/j.ajog.2019.07.031 (PMC7471839; doi:10.1016/j.ajog.2019.07.031)
Supplement: Appendix A [file mmc1.pdf]

UKCTOCS
United Kingdom Collaborative Trial of Ovarian Cancer Screening

PELVIC ULTRASOUND SCAN RESULTS

First Name ________________________ **Surname** ________________________ **Volunteer Ref No** ______________________

Date __________/_________/____________ **Ultrasonographer** ____

Please tick the relevant box

Mode of scan ☐ TRANSABDOMINAL ☐ TRANSVAGINAL ☐ BOTH **Latex allergy** ☐ YES ☐ NO

Period in last year ☐ YES ☐ NO **Date of last period (if within the last year)** _____/__________/__________

Type of HRT if used ☐ ESTROGEN ☐ PROGESTOGEN ☐ COMBINED CYCLICAL (CONVENTIONAL) ☐ COMBINED CONTINUOUS (NO BLEED) ☐ TIBOLONE ☐ OTHER

Hysterectomy ☐ **Previous oophorectomy** ☐ NONE ☐ LEFT OOPHORECTOMY ☐ RIGHT OOOPHORECTOMY

DETAILS OF OVARIAN SCAN

	LEFT OVARY / ADNEXA	RIGHT OVARY / ADNEXA
**VISUALISATION**<br>**MUST BE COMPLETED**	☐ SEEN<br>☐ NOT SEEN / GOOD VIEW<br>☐ NOT SEEN / POOR VIEW<br>☐ NOT SEEN / PREVIOUS OOPHERECTOMY	☐ SEEN<br>☐ NOT SEEN / GOOD VIEW<br>☐ NOT SEEN / POOR VIEW<br>☐ NOT SEEN/ PREVIOUS OOPHERECTOMY
**If ovary not seen, reason**	☐ BOWEL ☐ FIBROIDS<br>☐ PELVIC VARICOSITIES<br>☐ OTHER	☐ BOWEL ☐ FIBROIDS<br>☐ PELVIC VARICOSITIES<br>☐ OTHER
**OVARIAN DIMENSIONS**	mm mm mm	mm mm mm
**Morphology**<br>**MUST BE COMPLETED IF OVARY SEEN**<br>(If COMPLEX please write description of findings in abnormalities notes box & complete other side of this form. Also enter reference number at top of page)	☐ NORMAL<br>☐ SIMPLE CYST<br>☐ COMPLEX MORPHOLOGY	☐ NORMAL<br>☐ SIMPLE CYST<br>☐ COMPLEX MORPHOLOGY
	**If midline mass, please enter under left or right adnexa and describe below**<br>**If longstanding UNCHANGED complex morphology which has been previously investigated on UKCTOCS screening and is being managed conservatively, please fax form to Susan Davies on 0207 380 6929 for data entry**	
**Number of Cysts**<br>**(more than 1 cyst = complex morphology)**		
**Ovary mobile?**	☐ YES ☐ NO	☐ YES ☐ NO

Max double endometrial thickness ___________ **mms** **Fluid POD or ascites (max vertical diameter)** ____________ **mms**

Details of abnormalities: **Referred in view of incidental findings** ☐ YES ☐ NO

Type of Image Record ☐ NONE ☐ DISK ☐ PHOTO ONLY ☐ BOTH DISK AND PHOTO

***Result Classification /Recommended Action Must Be Completed At Time Of Scan**

☐ NORMAL hence ROUTINE SCREENING ☐ UNSATISFACTORY hence REPEAT LEVEL 1 SCAN

☐ ABNORMAL therefore LEVEL 2 / SURGERY **(If any other option required, contact Susan Davies on 0207 380 6913)**

- **As defined by protocol.**

Entered Signature Date

Checked Signature Date

October 2005

UKCTOCS
United Kingdom Collaborative Trial of Ovarian Cancer Screening

Signature____ Date ________________________

DETALLS OF ANY OVARIAN / ADNEXAL LESION DETECTED **VOLUNTEER REF (Please enter)**____________________

	LEFT OVARY/ADNEXA	RIGHT OVARY/ADNEXA
**Cyst dimensions**	mm mm mm	mm mm mm
**Cyst wall thickness**	mm	mm
**Cyst wall structure**	☐ SMOOTH ☐ IRREGULAR	☐ SMOOTH ☐ IRREGULAR
**Fluid in cyst**	☐ANECHOIC ☐ RANDOM ECHOGENICITY<br>☐UNIFORM ECHOGENICITY	☐ANECHOIC ☐ RANDOM ECHOGENICITY<br>☐UNIFORM ECHOGENICITY
**Cyst structures**	☐SEPTAE ☐ PAPILLATIONS	☐SEPTAE ☐ PAPILLATIONS
**Maximum septa thickness**	mm	mm
**Size of largest papillation**	mm	mm
**Solid areas**	☐ Yes ☐ No	☐ Yes ☐ No
**Overall impression of lesion**<br><br>(Classification using International Ovarian Tumour Analysis criteria)<br><br>**MUST BE COMPLETED IN THE PRESENCE OF AN OVARIAN LESION**	☐ Unilocular cvst<br>☐ Unilocular solid<br>☐ Multilocular cvst<br>☐ Multilocular solid<br>☐ Solid	☐ Unilocular cvst<br>☐ Unilocular solid<br>☐ Multilocular cvst<br>☐ Multilocular solid<br>☐ Solid

DOPPLER STUDY OF ABNORMAL AREA

**Doppler performed**	☐ YES ☐ NO
**Presence of colour signal**	☐ YES ☐ NO
**Location of colour signal**	☐ SEPTAE ☐ WALL<br>☐ SOLID AREA ☐ PAPILLATIONS<br>☐ OTHER
**Lowest RI measured**	
**Lowest PI measured**	
**Peak systolic velocity**	

Findings suggestive of ☐ DERMOID CYST ☐ ENDOMETRIOTIC CYST

Were scan images reviewed at CC? ☐ YES ☐ NO

Person at CC with whom results discussed ____

Suggested management ☐ AS RECOMMENDED ☐ OTHER **(if other, please enter details in notes)**

NOTES

October 2005
