# Supplementary material for: Serum HE4 and diagnosis of ovarian cancer in postmenopausal women with adnexal masses
Source: Am J Obstet Gynecol. 2020 Jan;222(1):56.e1–56.e17. doi: 10.1016/j.ajog.2019.07.031 (PMC7471839; doi:10.1016/j.ajog.2019.07.031)
Supplement: Appendix B [file mmc2.pdf]

Appendix 2

Multiple Imputation details

The multiple imputation (MI) paradigm was used to account for the missing values in the ovary/lesion volume and morphology data, as it was considered important to use all available cancers given the low event rate. Specifically, a MI procedure was used that filled in missing values in multiple variables iteratively by using chained equations - defined as a sequence of univariate imputation models fully conditional on the other included imputation variables. This approach is much more flexible than a multivariate normal imputation model, allows for complex, customised equations and accepts arbitrary patterns of missingness. Locularity, for left and right ovary, were separately imputed with a multinomial logistic model (none/uni/multilocular) and the results subsequently combined into the simpler locularity term previously described. Similarly, for DV, for each ovary, each dimension was first fully imputed in the log scale, and the combined volume variable created post imputation. In total 20 imputation sets were created and all 20 sets were used in producing an overall risk prediction model using Rubin's Rules.[24] This was true also for all the subset models that relied upon imputed data.

Firth logistic regression

The penalty term included into the likelihood in Firth logistic regression was originally conceived as a way of dealing with 'separation' in logistic models whereby estimates tend to infinity. However, the method can also be utilised to reduce the general bias prevalent in

standard ML logistic regression when the event rate is small. Note, that this form of penalty has a different goal to the ridge and lasso penalty, which aims to minimise prediction error by deliberately including downward bias in the model estimates.

Ultrasound morphology data

The *presence of a solid component* and *locularity we*re coded in numerical terms (e.g. 0, 1, 2) and a coefficient estimated for each non-reference category.
